# Regenerative Therapy at the Crossroads: From Cell-Based to Cell-Free Precision Medicine

**DOI:** 10.3390/bioengineering13050515

**Published:** 2026-04-29

**Authors:** Kandasamy Nagarajan ArulJothi, Ramya Lakshmi Rajendran, Byeong-Cheol Ahn, Prakash Gangadaran

**Affiliations:** 1 Department of Genetic Engineering, College of Engineering and Technology, SRM Institute of Science and Technology, Potheri, Kattankulathur, Chengalpattu 603203, Tamilnadu, India; aruljotn@srmist.edu.in; 2 Department of Nuclear Medicine, School of Medicine, Kyungpook National University, Daegu 41944, Republic of Korea; ramyag@knu.ac.kr; 3 Cardiovascular Research Institute, Kyungpook National University, Daegu 41944, Republic of Korea; 4 BK21 FOUR KNU Convergence Educational Program of Biomedical Sciences for Creative Future Talents, Department of Biomedical Sciences, School of Medicine, Kyungpook National University, Daegu 41944, Republic of Korea; 5 Department of Nuclear Medicine, Kyungpook National University Hospital, Daegu 41944, Republic of Korea

## 1. Opening Perspective: A Field in Transition

The study of regenerative medicine is an ongoing revolution in modern healthcare, with the aim of restoring the function of a diseased organ or a system through repair, replacing or regenerative modes, as opposed to conventional treatments that merely treat symptoms. Historically, regenerative medicine has employed cell-based therapies, involving stem cells, tissue engineering, grafting, and bioprinting, to restore the function of a diseased organ. However, safety, immune compatibility, tissue rejection, handling and storage, and regulatory issues remain key challenges in the clinical translation of cell-based regenerative medicine [1]. While the field of regenerative medicine stands at the pivotal phase of transition, emerging evidence suggests that the effect of regenerative medicine is not solely based on the cells, but their secreted factors. The concept of cell-free regenerative medicine has gained considerable attention, where exosomes, miRNAs and other secreted factors confer the desired action without the involvement of live cells and their associated risks.

The advent of genomics, system biology and artificial-intelligence-driven treatment options are reshaping how regenerative medicine can be engineered and delivered. In particular, regenerative strategies are being tailored with molecular and genetic profiles to overcome adversity and rejection. The evolution of regenerative medicine promises precise, controllable, and personalized modality, where the “cell-based to cell-free” transition marks the beginning of the precision clinical era [1,2].

## 2. The Rise and Limitations of Cell-Based Therapies

Modern regenerative medicine is built on the reality that live cells can be used as therapeutic agents. They have replaced the need for tissue grafting or replacement with intelligent biological systems in cell-based regenerative medicine. The use of autologous cells in transplantation and tissue engineering has significantly reduced immune rejection and the use of immunosuppressants by patients [3]. In recent years, hematopoietic stem cells, engineered mesenchymal stem cells (MSCs), and engineered immune cells have opened the frontiers of clinical applications. These approaches provide compelling evidence that damaged tissues can be restored or repaired by integrating functional self-renewing cells into the host systems [4].

MSCs have received widespread research attention due to their multipotent nature, where they can differentiate into osteoblasts, chondrocytes, and adipocytes, making them an ideal tool for tissue repair in conditions related to musculoskeletal and cardiovascular frameworks. MSCs can suppress T-cell proliferation and activate T-regs, making them an ideal candidate to treat autoimmune conditions and chronic inflammations [5,6,7]. MSCs have an advantageous property, tumor tropism, which refers to the ability to migrate to the tumor cells, and are considered to be autonomous cellular machines. Due to their tumor tropism, MSCs have been engineered to carry genetically modified payloads of interleukins from TRAILs and have been used as a hybrid bionic system to carry and precisely deliver the drugs to the tumor site.

The concurrent rise of cell-based therapies and genetic engineering also paved the way for personalized healthcare, since cells can be engineered to correct defects before administering them in the host. CRISPR-Cas9 technology was successfully applied in induced pluripotent stem cells (iPSCs) to fix errors before transplanting cells. Genetically engineered Chimeric Antigen Receptor T-cell (CAR T-cells) could evade host cell inhibitory molecules to more effectively fight cancer.

Despite these advantages, cell-based therapies face significant challenges and limitations in clinical translation. Prominent limitations in cell-based therapies include impermeability to solid tumors and the blood–brain barrier, inconsistency among batches, manufacture failure, and high manufacturing costs.

Notably, in MSC therapy, long-term survival and integration into cell systems remain significant challenges, with post-administration poor homing efficiency often recorded as less than 5%. Additionally, the rapid aging of stem cells during in vitro preparatory phases and cryopreservation of cells diminishes their stemness and effector potential, leading to treatment failure. In vitro handling also poses a risk of genetic mutations due to continuous cultivations and inadvertent microbial contamination, leading to health risks in the hosts. There is a risk of the altered differentiation of injected cells occurring in the host, which may lead to inappropriate cell types and a complicated microenvironment. Oxidative stress in the host microenvironment may eliminate the injected stem cells, leading to treatment failure or decreased efficiency [6]. The lack of safety regarding potential stem cell transformation into malignant forms remains a major concern in cell-based regenerative therapy.

Regulatory limitations also negatively impact cell-based therapies. The use of embryonic stem cells has raised serious ethical concerns in many countries as it leads to the destruction of human embryos. Adult stem cell usage also had complex issues regarding informed consent, which requires a thorough understanding of the procedures by the donors, since cells and genetic materials have long-term effects and could be subject to misuse in the future [5].

## 3. Emergence of Cell-Free Therapeutics

As the limitations of cell-based therapies are apparent, a fundamental question remains: Are the cells themselves therapeutic agents or are they just factories that produce active therapeutics? This shift led to the new paradigm of cell-free regenerative therapies that exploited the functional factors released by the cells without relying on the cells themselves [8]. A key aspect of this transition is the recognition that the regeneration potential attributed to the transplanted cells is mediated through their secretome. Bioactive compounds—such as exosomes, cytokines, growth factors, and regulatory RNAs—have been shown to coordinate key processes, including inflammation, angiogenesis, and tissue repair. By isolating and delivering these crucial factors directly in therapeutic approaches, cell-free strategies aim to replicate the regenerative benefits of whole cells while minimizing adverse effects. Cell-free strategies offer various advantages, including improved safety profiles, reduced immunogenicity, and tumorigenicity [9]. Cell-free products can be characterized, quantified, and manufactured consistently, where optimization and scalability are also plausible for real-life applications. Importantly, tailored forms of vesicles and a cocktail of molecular factors with defined functions can be created using bioengineering tools with secretome-based regenerative approaches [10].

While stem cells are attractive for regenerative applications, extracellular vesicles (EVs) can evade many barriers that stem cells face. EVs are non-replicating bodies and therefore avoid the risks of neoplasms, as opposed to stem cell administration. The autologous EVs could escape immune rejection. The lipid bilayer of EVs provides protection and stability to therapeutic cargo in the bloodstream, favoring the crossing of the blood–brain barrier and transfer of therapeutic miRNA or proteins directly into the cell. EVs have exhibited promising potential in cardiomyocyte regeneration and increase angiogenesis post myocardial infarction [11].

## 4. Precision Regenerative Medicine: Convergence of Technologies

Precision regenerative medicine emerges at the intersection of multi-disciplinary technological advancements, where the convergence of genomics, gene editing, biomaterials, bioengineering, and artificial intelligence is reshaping therapeutic strategies. An integrative approach combining these technologies makes tailored, precision delivery and treatments possible in regenerative medicine. In traditional stem cell technology, the development of iPSCs aid in making patient-specific organoid models for drug testing and predicting the prognosis of the patients based on their genetic profiles. CRISPR–Cas9 gene editing technology enables the correction of altered genes in stem cells, which restores the original function of organs. Bioprinting and bioink technologies make the artificial construction of complex microscale tissue structures for grafting purposes with enhanced vascularization. Biomaterials have been used in extracellular matrix (ECM) construction following the introduction of hydrogels, bioceramics, and nanotechnology. The latest advancements in microfluidics include the assembly of an organ-on-a-chip by introducing live cells onto slides to mimic organs, for use in drug testing and as disease models. Finally, artificial intelligence is used to optimize and construct scaffolds, predict properties using neural network models and assess genome data for personalized therapies [12]. Together, the convergence of stem cells, gene editing, advanced materials, and artificial intelligence driven modeling transforms regenerative medicine into a dynamic, personalized organ restoration technology [13].

## 5. Remaining Challenges and Translational Gaps

Despite recent advancements, several critical challenges remain in the clinical translation of cell-free regenerative therapy. The variations seen during the isolation and purification of EVs or effector molecules destined for regenerative therapy leads to varied therapeutic outcomes. The precise localization and delivery of cell-free products, as well as confining them at the site of injury to elicit the desired action, remain technically difficult. The heterogenous profile of the secretome makes it difficult to characterize the active ingredients associated with the effector function [14]. Current regulatory protocols in different countries are still evolving and in the formative stages of dealing with acellular products. Although the challenges faced by cell-free regenerative therapy are unique, there is substantial progress to be made before durable patient care is attainable. There is a major lack of understanding surrounding how biological variables such as age and gender influence the outcome of treatment [15]. Inadequate technology for monitoring genetic and epigenetic aberrations leads to increased safety concerns. The degradation of employed biomaterials at a perfectly synchronized rate with the regenerated tissue is a persistent engineering flaw hat warrants substantial experimental validations to address. The lack of hospital infrastructure to implement the protocols required in regenerative procedures remains a major logistical challenge [16].

## 6. Future Directions: Beyond the Crossroads

The future of regenerative medicine depends on not just what replaces the cells, but how rationally the biological signals are engineered, delivered, and integrated into the host system. Three-dimensional bioprinting is developing into 4D/5D bioprinting, where the new dimension is time and the biomaterials respond to external stimuli such as pH, light, and temperature. They can even respond to cell-stimulated responses to expand ECM shaping post fabrication, in order to mimic natural processes [17]. Smart biomaterials can be standardized and potentially delivered via a biocompatible matrix (like a patch or gel) for the local treatment of digital ulcers and skin lesions. For the exosomes, miRNA released from the MSCs can modulate gene expression through histone modification, providing a promising strategy to control gene expression and avoid unintended consequences. Moreover, the advent of synthetic biology presents smart sensor-based systems, to recognize cues and deliver therapeutics such as insulin or cytokine when needed in specified organs or tissues. The development of externally controllable gene circuits marks a transformative advance at the intersection of gene editing technologies and regenerative medicine, enabling precise spatiotemporal regulation of cellular functions [18]. The integration of artificial intelligence, multi-omics profiling, engineered extracellular vesicles, and advanced biomaterial design platforms are already dominant in the healthcare industry. In the context of regenerative medicine, they may be used to predict patient responses based on their molecular and genomic profiles and can even be used to optimize and construct the regenerative materials required for precision delivery and treatment [19,20].

## 7. Concluding Insights

Regenerative medicine stands at a critical juncture where advanced technologies such as 4D/5D bioprinting, biomaterials, secretome, gene editing and artificial intelligence face significant challenges. While these advances, along with regenerative technology, converge to offer unprecedented outcomes in precision medicine and organoid fabrication, the field faces several bottlenecks related to ethical concerns, scalability, and the lack of standardization. Moving forward, the successful clinical translation of regenerative medicine can be achieved not through isolated discoveries, but through interdisciplinary collaboration between biologists, engineers, and clinicians. Together, through comprehensive collaborations and rigorous ethical oversights, the potential of regenerative medicine can be translated into stable patient outcomes.

## References

[1] Hoang V.T., Nguyen Q.T., Phan T.T.K., Pham T.H., Dinh N.T.H., Anh L.P.H., Dao L.T.M., Bui V.D., Dao H.N., Le D.S. (2025). Tissue Engineering and Regenerative Medicine: Perspectives and Challenges. MedComm.

[2] Jian A., Zhao G., Wang Y., Wang S., Li N. (2025). Watershed Year of Cell and Gene Therapy (CGT): A Review of 2024 CGT Approvals. Cancer Lett..

[3] Xia L., Qin J., Ye S. (2025). Addressing the Challenges of Immunogenicity, Heterogeneity and Tumorigenicity in the Clinical Translation of Human Embryonic Stem Cells. Stem Cell Res. Ther..

[4] Wang J., Deng G., Wang S., Li S., Song P., Lin K., Xu X., He Z. (2024). Enhancing Regenerative Medicine: The Crucial Role of Stem Cell Therapy. Front. Neurosci..

[5] Hussen B.M., Taheri M., Yashooa R.K., Abdullah G.H., Abdullah S.R., Kheder R.K., Mustafa S.A. (2024). Revolutionizing Medicine: Recent Developments and Future Prospects in Stem-Cell Therapy. Int. J. Surg..

[6] Mei R., Wan Z., Yang C., Shen X., Wang R., Zhang H., Yang R., Li J., Song Y., Su H. (2024). Advances and Clinical Challenges of Mesenchymal Stem Cell Therapy. Front. Immunol..

[7] Velikova T., Dekova T., Miteva D.G. (2024). Controversies Regarding Transplantation of Mesenchymal Stem Cells. World J. Transplant..

[8] Jarrige M., Frank E., Herardot E., Martineau S., Darle A., Benabides M., Domingues S., Chose O., Habeler W., Lorant J. (2021). The Future of Regenerative Medicine: Cell Therapy Using Pluripotent Stem Cells and Acellular Therapies Based on Extracellular Vesicles. Cells.

[9] Rajendran R.L., Mahajan A.A., Muthu S., Rajappan Chandra S.K., Gangadaran P., Ahn B.C. (2026). Global Research Trends in Extracellular Vesicle–Based Therapy for Regenerative Medicine: A Bibliometric Analysis (2014–2024). Bioengineering.

[10] Napolitano F., Giudice V., D’Esposito V., Prevete N., Scala P., de Paulis A., Selleri C., Formisano P., Rossi F.W., Montuori N. (2025). Cell-Free Regenerative Medicine: Identifying the Best Source of Mesenchymal Stem Cells for Skin Therapy in Systemic Sclerosis. Front. Cell Dev. Biol..

[11] Rai A., Claridge B., Lozano J., Greening D.W. (2024). The Discovery of Extracellular Vesicles and Their Emergence as a Next-Generation Therapy. Circ. Res..

[12] Jalali S.N., Fathi Z., Mohebbi S. (2025). Innovative Platforms in Regenerative Medicine: Bridging Research and Clinical Solutions. Jentashapir J. Cell. Mol. Biol..

[13] Shi Y., Zhang L., Chen M., Calle E.A., Langer R. (2025). Regenerative Engineering: Evolution and Its Modern Significance. Regen. Eng. Transl. Med..

[14] Ganesan O., Kiwanuka H., Hamaguchi R., Orgill D.P. (2025). A Review of Regenerative Medicine and Tissue Engineering with a Focus on Wound Healing and Anti-Aging. Front. Surg..

[15] Yu S., Kong D., Lu B., Pan Y., Zeng Z., Fu Y., Zhao Z., He K., Tang R., Xia Q. (2025). Mesenchymal Stem Cell-Derived Exosomes as Cell-Free Therapeutics: Mechanistic Insights and Engineering Strategies for Liver Disease Treatment. Stem Cell Res. Ther..

[16] Serrano D.R., Juste F., Anaya B.J., Ramirez B.I., Sánchez-Guirales S.A., Quispillo J.M., Hernandez E.M., Simon J.A., Trallero J.M., Serrano C. (2025). Exosome-Based Drug Delivery: A Next-Generation Platform for Cancer, Infection, Neurological and Immunological Diseases, Gene Therapy and Regenerative Medicine. Pharmaceutics.

[17] Idnani R., Khan S. (2026). Engineering the Future of Organ Transplantation: A Comprehensive Review of 3D Bioprinting Advances for Organ Bioengineering. Bioprinting.

[18] Mohammadi A., Heydari M.A., Jamalpoor Z. (2025). Genetic Engineering Frontiers in Cell Manipulation-Based Tissue Engineering: A Comprehensive Review. BioImpacts.

[19] Gabaran S.G., Ghasemzadeh N., Rahnama M., Karatas E., Akbari A., Rezaie J. (2025). Functionalized Exosomes for Targeted Therapy in Cancer and Regenerative Medicine: Genetic, Chemical, and Physical Modifications. Cell Commun. Signal..

[20] Nosrati H., Nosrati M. (2023). Artificial Intelligence in Regenerative Medicine: Applications and Implications. Biomimetics.

